# Adherence to Physical Activity Recommendations in the Adult Population of Jazan Region

**DOI:** 10.7759/cureus.23481

**Published:** 2022-03-25

**Authors:** Ahmed Wafi, Abdulrahman Aqeel, Basem Zogel, Ayman Shami, Abdullah Alharthi, Abdulelah Alameer, Abdulrahman Kulaybi, Ahmed Sumayli, Youmna Abutalib, Abdulrahman Alessa, Abdulaziz H Alhazmi

**Affiliations:** 1 Physiology, Jazan University, Jazan, SAU; 2 Medicine and Surgery, Jazan University, Jazan, SAU; 3 Microbiology, Jazan University, Jazan, SAU; 4 Medical Research Center, Jazan University, Jazan, SAU

**Keywords:** obesity, jazan, kingdom of saudi arabia (ksa), body mass index: bmi, physical activity

## Abstract

Background

Physical activity (PA) is an important determinant of health because of its role in preventing multiple chronic diseases. A better understanding of the relationship between PA and factors that promote or hinder PA is necessary for adherence to PA recommendations. This study aimed to estimate the proportion of adults of the Jazan region of Saudi Arabia adhering to PA recommendations and to examine the effects of socio-economic factors on meeting these recommendations.

Method

The official short form of the International Physical Activity Questionnaire (IPAQ) was used to assess PA. Participants aged 17-74 years (n = 709) were categorized into active or inactive categories. Independent associations between PA categories and socio-economic factors were explored using binary logistic regression.

Results

The proportion of the adults of the Jazan region who were inactive, i.e., not adhering to the PA recommendations, was 58.3%. Those with body mass index >30 kg /m^2^ (OR = 0.51; 95%CI: 0.32-0.84) were less likely to be more active than those with normal weight. Participants who rated their health as very good (OR = 0.68; 95% CI: 0.49-0.96) and good (OR = 0.39; 95% CI: 0.22-0.70) were less likely to be more active than those who rated their health as excellent.

Conclusions

More than half of the adult population of the Jazan region did not adhere to the PA levels necessary to promote health and prevent chronic diseases. Such a high prevalence of low PA is a major health problem. Thus, efforts are warranted to promote PA in the Jazan region, particularly among obese female adults. High self-perceived health was an important correlate of PA among the adult population of Jazan. Because of the wide range of physical activity levels among population subgroups, a social-ecological approach for physical activity promotion may be warranted.

## Introduction

Current physical activity (PA) guidelines from the American Heart Association and American College of Sports Medicine acknowledge the importance of health-enhancing PA in the primary and secondary prevention of several chronic diseases [1-3]. In part, the prevention of chronic health problems depends on controlling predisposing risk factors, including physical inactivity, which is considered an independent risk factor for several chronic diseases such as coronary heart disease (CHD), diabetes mellitus, obesity, osteoporosis, and hypertension [4,5].

Worldwide, physical inactivity contributes to approximately 9% of premature mortality [6]. According to the World Health Organization (WHO), physical inactivity is considered the fourth leading cause of non-communicable chronic diseases after high blood pressure, smoking, and high blood glucose [7]. More than a quarter (27.5%) of all adults do not meet recommendations for aerobic exercise as outlined in the 2010 Global Recommendation on Physical Activity for Health [8,9]. Furthermore, global estimates have also shown that physical inactivity is responsible for 22% of ischemic heart disease and 10-16% of diabetes mellitus, breast, colon, and rectal cancer [8]. On the other hand, regular PA has been shown to lower the risk of numerous diseases, including cardiovascular diseases, type 2 diabetes, osteoporosis, and certain cancer types [3,4,6]. Therefore, PA monitoring is becoming a public health priority.

Previous reports indicate that most Saudi adults do not meet the recommended PA levels necessary for health promotion and disease prevention [10,11]. Importantly, physical inactivity in Saudi Arabia appears to be a major risk factor for CHD [12-14]. The proportion of Saudi adults at risk for CHD due to physical inactivity is much higher than those at risk due to other CHD risk factors, such as high blood pressure, high blood cholesterol, obesity, and cigarette smoking [10]. Physical inactivity is not only a predisposing factor for chronic diseases but also imposes a huge economic burden. In 2013, it was estimated that the direct and indirect costs of physical inactivity in Saudi Arabia represent approximately 1.71% of total health care costs [5].

Although previous physical activity assessments revealed that physical inactivity is prevalent among the Saudi population [15-18], most of these assessments had primarily focused on leisure-time PA, which reflects the adherence to the recommendation of intentional exercise with a little emphasis on other components of PA that may take place during the course of transport, work, etc. An important feature of the International PA Questionnaire (IPAQ) is that it assesses reliable and valid information about PA at several intensity levels and across several domains, including home, at work, during transportation, and during leisure time [19]. 

To design and implement interventions to promote PA, it is necessary for physicians and public health professionals to be aware of the least active groups in society and those who would benefit most from increasing their PA levels. Therefore, it is important to understand how sociodemographic factors influence PA. This study aimed to assess the adherence of adults from the Jazan region of Saudi Arabia to the PA guidelines and assess the effect of socio-economic factors, body mass index (BMI), and self-perceived health on PA levels.

## Materials and methods

Study design and population 

The study design was an observational, cross-sectional, self-administered electronic survey targeted at adults (aged between 17 and 74 years) in the Jazan region, which is one of the thirteen regions comprising Saudi Arabia. It is located in the southwestern part of Saudi Arabia, with more than 1.5 million residents.

Sampling procedure

Using power analysis, we calculated that we needed 520 participants to achieve statistical significance (p<0.05) at a power of 0.8, assuming a non-response rate of 25%. However, 709 participants were included in the study to further increase the study power.

The participants were invited to fill out an online survey randomly distributed through social media platforms (WhatsApp, Meta Platforms, Inc., Menlo Park, California, United States (US); Twitter, Twitter, Inc., San Francisco, California; Facebook, Meta Platforms, Inc., Menlo Park, California, US) using the snowball sampling recruitment method. The information sheet and consent form were included on the first page of the survey created by Google Forms (Google LLC, Mountain View, California, US).

Out of 1416 people who were initially contacted, we were able to get a response from 887 persons (about a 63% response rate). Out of the 887 persons, 709 filled the minimum information necessary for the analysis.

The Jazan University Scientific Research Ethics Committee approved the protocol for this study with reference number REC42/1/152. The collected data were held anonymously without any indication of any personal information. The inclusion criteria were Saudi adults aged between 17 to 74 years residing in the Jazan region, Saudi Arabia. The exclusion criteria included non-Saudis, responders younger than 17 years or older than 74 years, Saudis who were not from the Jazan region, and those who did not report correctly.

Assessment of PA

The official Arabic short version of the IPAQ was used to assess PA [12]. The short version has been shown to have an acceptable test-retest reliability of 0.8 and criterion validity compared with accelerometers (rho = 0.3) [19]. The IPAQ assesses the PA levels by asking each individual the number of days per week (how often) and the average time in minutes (how long) he/she has been active at moderate intensity, vigorous intensity, and walking. An average metabolic equivalent (MET) was assigned to each intensity according to the IPAQ scoring protocol: MET scores were 3.3, 4.0, and 8.0 METs for walking, moderate intensity, and vigorous-intensity PA, respectively. The data were scored according to the IPAQ scoring protocol available on the IPAQ website. Based on the current PA guidelines, adults should stay active on most days, preferably all days of the week, accumulating 30 minutes of moderate-intensity PA [1]. This corresponds to 600 MET minutes per week (30 minutes per day for five days equivalent to 4.0 MET) on the IPAQ scoring system, which represents the lowest limit for the moderate-intensity PA category. Additionally, the moderately active cut-off limit allows a person to perform vigorous-intensity PA for three days per week for 20 minutes each day (20 minutes per day for three days equivalent to 8.0 MET = 480 MET minutes per week). The high PA category includes either doing vigorous-intensity PA for three days or more per week, accumulating 1500 MET minutes or 3000 MET minutes per week with any combination of walking, moderate, or vigorous-intensity PA.

Because PA is measured across all domains by the IPAQ, and the PA guidelines are mainly based on leisure-time PA, the moderate PA category cut-off limit is considered the absolute minimum of PA for some health benefits [20]. According to the IPAQ scoring protocol, activity frequency and duration are used to classify subjects into low, moderate, and high PA levels. However, since we aimed to identify population proportion that adhered to PA recommendation, PA was dichotomized into either inactive (low PA category) or active (moderate or high PA categories) based on whether the cut-off for reaching the moderately active category was met [21]. Individuals who did not report frequency and time in one intensity category were excluded from the analysis as the PA level could not be quantified.

Socio-economic factors and BMI

Participants were divided into three age groups: 17-34, 35-54, and 55-74 years. Educational level was categorized as doctorate/masters, bachelor, higher diploma, basic school education (primary, intermediate, and secondary). Employment was categorized as employed, student, retired, and unemployed. The participants were divided according to the monthly income into four groups: <Saudi Riyal (SAR) 5000, SAR 5001-10000, SAR 10001-20000, and >SAR 20000. The participants' marital status was categorized into married, single, divorced, and widowed. The participants were classified as current smokers, previous smokers, and never smokers. The number of children was categorized into none, one or two, and three or more. The participants were also asked to rate their current overall health status as follows: excellent, very good, good, satisfactory, or poor. BMI was calculated by dividing body weight by height squared (kg/m^2^). A BMI of 18.5-24.9 was categorized as normal weight, 25-29.9 was categorized as overweight and ≥30 was categorized as obese [22].

Statistical analyses

Statistical analyses were performed using the IBM SPSS Statistics for Windows, Version 23.0 (Released 2015; IBM Corp, Armonk. New York, US). Frequencies and percentages were calculated for both inactive and active PA categories and for each socio-economic factor and BMI. Bivariate relationships between PA categories and socio-economic factors, BMI, and self-perceived health were tested using Chi-squared analysis. The effect of each independent variable on the categories of PA was assessed by binary logistic regression.

The OR and their 95%CI were calculated against the following reference categories: sex, female; age, 55-74 years; BMI, normal weight; education, basic education; employment status, unemployed; monthly income, the highest income group; marital status, married; number of children, more than three; smoking habits, smokers; and those with an excellent self-rated health condition.

## Results

Adherence to the PA recommendation

Table 1 shows the frequency distribution of the study participants. Compared to the official statistics of Saudis [23], there was an under-representation of women in this study (37.5%) compared to Saudi females in the Jazan region (49.4%). A total of 413 (58.3%) respondents were classified as physically inactive, i.e., not adhering to the PA recommendations.

**Table 1:** Frequency distribution of the study participants BMI: body mass index; SAR: Saudi Riyal

Variable	N	%
Physical activity		
Inactive	413	58.3
Active	296	41.7
Sex		
Male	443	62.5
Female	266	37.5
Age (years)		
17–34	532	75.0
35–54	157	22.1
55–74	20	2.8
BMI		
Normal	374	52.8
Overweight	208	29.3
Obese	127	17.9
Education		
Basic	176	24.8
Diploma	56	7.9
Bachelor	453	63.9
Graduate	24	3.4
Employment status		
Student	381	53.7
Unemployed	62	8.7
Employed	243	34.3
Retired	23	3.2
Monthly income (SAR)		
< 5K	434	61.2
5K–10K	108	15.2
10K–20K	144	20.3
> 20K	23	3.2
Marital status		
Single	418	59.0
Married	276	38.9
Divorced	12	1.7
Widowed	3	0.4
Number of children		
0	466	65.7
1–2	89	12.6
> 3	154	21.7
Smoking status		
Non-smoker	577	81.4
Smoker	95	13.4
Former smoker	37	5.2
Self-rated health condition		
Poor or satisfactory	17	2.4
Good	81	11.4
Very good	308	43.4
Excellent	303	42.7

The highest proportion of participants not adhering to the recommendations was found in the non-smoking group, where 82.8% were categorized as inactive. Young adults (17-34 years) had the second-highest proportion (72.2%). Significant variations between PA categories were observed by sex, age, BMI, employment, marital status, number of children, and self-rated health condition, but not by education level, monthly income, and smoking habits (Table 2).

**Table 2:** Sample characteristics according to PA category *Significant difference BMI: body mass index; SAR: Saudi Riyal

	Inactive		Active		P
Variable	N	%	N	%	
Sex					0.027*
Male	244	59.1	199	67.2	
Female	169	40.9	78	32.8	
Age (years)					0.039*
17–34	298	72.2	234	79.1	
35–54	99	24.0	58	19.6	
55–74	16	3.9	4	1.4	
BMI					0.003*
Normal	203	49.2	171	57.8	
Overweight	119	28.8	89	30.1	
Obese	91	22.0	36	12.2	
Education					0.437
Basic	102	24.7	74	25.0	
Diploma	36	8.7	20	6.8	
Bachelors	258	62.5	195	66.9	
Graduate	17	4.1	7	2.4	
Employment status					0.011*
Student	207	50.1	174	58.8	
Unemployed	38	9.2	24	8.1	
Employed	148	35.8	95	32.1	
Retired	20	4.8	3	1.0	
Monthly income (SAR)					0.331
< 5K	244	59.1	196	64.2	
5K–10K	62	15.0	46	15.5	
10K–20K	91	22.0	53	17.9	
> 20K	16	3.9	7	2.4	
Marital status					0.009*
Single	222	53.8	196	66.2	
Married	180	43.6	96	32.4	
Divorced	9	2.2	3	1.0	
Widowed	2	0.5	1	0.3	
Number of children					0.026*
0	255	61.7	211	71.3	
1–2	60	14.5	29	9.8	
> 3	98	23.7	56	18.9	
Smoking					0.483
Non-smoker	342	82.8	235	79.4	
Smoker	52	12.6	43	14.5	
Former smoker	19	4.6	18	6.1	
Self-rated health condition					<0.001*
Poor or satisfactory	9	2.2	8	2.7	
Good	62	15.0	19	6.4	
Very good	190	46.0	118	39.9	
Excellent	152	36.8	151	51.0	
Total	413	100	296	100	

Influence of socio-economic factors 

A binary logistic regression was conducted to determine the impact of socioeconomic factors on the likelihood of participants being physically inactive (Table 3). Women appeared to be more likely to be classified as physically inactive, but the results did not reach significance (p=0.07). Only two variables significantly predicted the likelihood of being physically inactive: BMI and self-rated health condition. For BMI, the OR for those who were obese was 0.51 (95%CI: 0.32-0.84) indicating that they were less likely to be active compared to those with normal body weight. Respondents who self-reported their health condition as very good or good had decreased odds of being physically active (OR = 0.68; 95%CI: 0.49-0.96 and OR = 0.39; 95%CI: 0.22-0.70, respectively) compared with those who self-rated their health condition as excellent.

**Table 3:** Binomial logistic regression for the physical activity categories according to socio-economic factors BMI: body mass index; SAR: Saudi Riyal

			95% CI	
Variable	p	OR	lower	Higher
Sex				
Male	0.072	1.40	0.97	2.03
Female		1.00		
Age (years)				
17–34	0.739	0.765	0.157	3.72
35–54	0.805	0.833	0.194	3.58
55–74		1.00		
BMI				
Overweight	0.90	0.98	0.67	1.43
Obese	0.007	0.51	0.32	0.84
Normal		1.00		
Educational Level				
Diploma	0.534	1.260	0.608	2.613
Bachelor	0.409	1.173	0.803	1.714
Graduate	0.991	0.994	0.336	2.938
Basic		1.00		
Employment status				
Student	0.367	1.317	0.724	2.395
Employed	0.962	0.982	0.463	2.081
Retired	0.115	0.248	0.044	1.404
Unemployed		1.00		
Monthly income (SAR)				
< 5k	0.448	1.564	0.493	4.964
5k–10k	0.234	1.979	0.642	6.098
10k–20k	0.277	1.779	0.629	5.031
>20k		1.00		
Marital status				
Single	0.199	1.606	0.779	3.312
Divorced	0.745	1.280	0.290	5.662
					Widowed	0.899	1.177	0.095	14.582
Married		1.00		
Number of children				
0	0.40	0.70	0.30	1.63
1–2	0.23	0.65	0.33	1.30
>3		1.00		
Smoking status				
Non-smokers	0.441	0.828	0.513	1.337
Former smokers	0.510	1.316	0.581	2.979
Smokers		1.00		
Self-perceived health				
Poor or satisfactory	0.99	1.01	0.36	2.86
Good	0.002	0.39	0.22	0.70
Very good	0.03	0.68	0.49	0.96
Excellent		1.00		

Sex-specific analyses 

Only variables significantly contributing to the physical inactivity prediction were investigated. The self-rated health condition was associated with PA levels among men, while BMI was associated with PA levels among women (Table 4). Men who self-reported their health condition as very good and good were less likely to be physically active than those whose self-rated health condition was excellent (OR = 0.60; 95% CI: 0.39-0.93 and OR = 0.32; 95% CI: 0.15-0.70, respectively). Obese women were much less likely to be physically active than normal-weight women (OR = 0.06; 95%CI: 0.12-0.39).

**Table 4:** Analyses of physical activity categories according to sex BMI: body mass index

Variable	P	OR	95% CI		
			Lower		Upper
Female sex					
BMI					
Overweight	0.61	0.83	0.41	1.68	
Obese	0.003	0.06	0.12	0.39	
Normal		1.00			
Self-perceived health					
Poor or satisfactory	0.22	3.40	0.49	23.73	
Good	0.61	0.78	0.29	2.07	
Very good	0.60	0.85	0.47	1.54	
Excellent		1.00			
Male sex					
BMI					
Overweight	0.62	1.12	0.70	1.80	
Obese	0.19	0.69	0.40	1.20	
Normal		1.00			
Self-perceived health					
Poor or satisfactory	0.52	0.61	0.14	2.70	
Good	0.005	0.32	0.15	0.70	
Very good	0.02	0.60	0.39	0.93	
Excellent		1.00			

## Discussion

Given the accepted validity of the short form IPAQ, reasonable sample size, and inclusion of several potential confounding factors, we feel that this result could be generalized to the adult population of the Jazan region regarding adherence to PA recommendation and the association between the PA categories and the studied factors.

During the past several decades, Saudi Arabia has witnessed substantial socioeconomic development and prosperity. This may contribute to such a high prevalence of physical inactivity among Saudi adults and a subsequent increase in lifestyle-related non-communicable diseases in the country including the Jazan region. The IPAQ has been used to evaluate PA in other large-scale population studies, such as the WHO survey of 51 countries [24]. However, as Saudi Arabia was not included in this study, a comparison with these results cannot be made. Nonetheless, compared with the prevalence of physical inactivity in the United Arab Emirates (43%) and in a regional Saudi study (40.6%) [12], our estimate in this study was higher (58.3%). This discrepancy can be partially explained by the fact that self-report of PA is influenced by a variety of factors, most notably over-reporting of PA time and intensity [25]. To our knowledge, only one study reported the prevalence of PA among the Jazan adult population (5%) [26]. However, this study did not use IPAQ to assess the PA, which may explain different estimates of PA profile compared with our results. National studies that assessed PA prevalence using the same tool in our study (IPAQ) suggested that 58% [27] and 64.4% [28] did not adhere to the current PA recommendation, close to our estimate of 58.3%. Given the wide variations in studies regarding sex, age range, inactivity criteria, and PA assessment, comparing physical inactivity profiles should be made with caution.

Significant variations between inactive and active PA categories were observed in terms of sex, age, BMI, employment, marital status, number of children, and self-perceived health condition. These findings suggest that the IPAQ is a valid and feasible instrument, which provides useful information for formulating a national strategy to recognize the target population most needing PA promotion strategies.

The present study revealed that inactive females were less than males, a finding that is not similar to what was previously reported [27,28]. While this finding could be explained by the fact that the IPAQ instrument includes moderate-intensity household activities likely to be performed by females, we believe that this finding is likely due to the low number of female participants in our sample.

This study demonstrated that the age group of 55-74 years exhibited a higher prevalence of physical inactivity than the other age groups. This finding appeared to be consistent with those of previous studies conducted nationally and internationally [15,29,30], which suggests a general pattern of a negative association between age and physical inactivity. The older age group is an important group to target with PA interventions as they can benefit most from increased PA [31,32]. A previous study on PA levels among Saudi males between the ages of 19 and 68 years reported a curvilinear relationship between age and physical inactivity, with inactivity prevalence being the highest in the age group of 40-49 years [33]. However, this study assessed leisure-type PA, which may partially explain the different results regarding physical inactivity rates and age.

Although people with high socioeconomic status (high income and high education) are frequently shown to report more leisure-time PA than those with low socioeconomic status [15,34,35], our results indicated that both income and educational level were not associated with physical inactivity. It appears that the association between PA and educational levels depends, in part, on the domain of PA assessed. Such association between PA and educational level is likely to be seen in the leisure-time PA domain, which was not evaluated in this study.

An inverse relationship between PA and BMI was observed in this study. Those with a BMI above 30, especially women, had the lowest proportion of reaching the cut-off limit for meeting the PA recommendation. This may indicate that physical inactivity, at least in part, contributes to obesity. However, it appears that the relationship between physical inactivity and obesity is an association rather than a causal relationship. The evidence for the role of PA in obesity prevention suggests only a weak association between a low level of physical inactivity and weight gain, and interventions with increased PA levels had a small effect on obesity [36]. While there is a need for evidence-based studies to see whether increasing PA is an effective measure to prevent obesity, it is encouraged, in the meantime, to follow the established PA recommendation advocating 30 minutes of daily moderate-intensity PA to prevent obesity.

Self-perceived health condition was an important determinant of PA, especially among males, which supports previous findings [37,38]. However, given the cross-sectional design of this study, it is difficult to determine whether PA improves self-perceived health or if those with high self-perceived health do more PA.

Taken together, our results indicated that PA levels differ substantially among societal groups; as such, potential PA interventions cannot be directed to a particular group. Several PA promotion strategies have not been successful, especially regarding the long-term maintenance of PA [39]. Socio-ecological intervention models in which external factors such as the physical environment are expected to have a relatively permanent effect [40].

The present study has strengths and limitations. The strengths include using a validated self-administered questionnaire covering PA domains. To our knowledge, this is the first study assessing the prevalence of physical inactivity among Jazan region adults using IPAQ. We also acknowledge several limitations in our study with the potential for bias in self-reported PA being a major limitation. In addition, the cross-sectional design of our study does not allow us to make an inference with regard to causality. The under-representation of women in this study might have influenced the PA estimate in the entire sample.

## Conclusions

In conclusion, this study found that 58.3% of the adult population in the Jazan region of Saudi Arabia do not adhere to the PA recommendation of at least 30 minutes of moderate-intensity PA on most, or preferably all, days of the week. This means that more than half of the adults in the Jazan region are not active enough to meet the recommended PA level. The main risk groups for physical inactivity were obese individuals with low self-perceived health.

As the PA level differs between populations according to socioeconomic factors, there appears to be a need for socio-ecological intervention to promote PA. Given the large variability in the prevalence of physical inactivity profiles among Saudi adults, future studies using objective measures such as accelerometers are needed to assess physical inactivity accurately. In addition, future studies that consider regional differences in terms of important variables that could affect the physical inactivity estimates are also needed.
